# Editorial: Updates on radiation-induced lymphopenia

**DOI:** 10.3389/fonc.2024.1448658

**Published:** 2024-07-02

**Authors:** Pim J. J. Damen, Steven H. Lin, Peter S. N. van Rossum

**Affiliations:** ^1^ Department of Radiation Oncology, Amsterdam UMC, Amsterdam, Netherlands; ^2^ Department of Radiotherapy, Erasmus MC Cancer Institute, University Medical Center Rotterdam, Rotterdam, Netherlands; ^3^ Department of Radiation Oncology, The University of Texas MD Anderson Cancer Center, Houston, TX, United States

**Keywords:** lymphopenia, radiotherapy, review, survival, immunotherapy

Radiotherapy is a commonly employed and effective treatment for cancer, seeking to achieve an optimal balance between tumor cell death and minimizing damage to healthy tissues. Radiation-induced lymphopenia (RIL) is a common side effect due to the high radiosensitivity of lymphocytes even at low doses (<1Gy), leading to their direct depletion (1). While the detrimental effects of radiotherapy on lymphocytes have been recognized since 1905, its influence on tumor control and survival has remained largely unclear until recently (2). Moreover, awareness and understanding of the prognostic effects of RIL remain limited in daily clinical practice.

Radiotherapy induces a substantial drop in lymphocyte levels during treatment, with the most significant decrease occurring in the first week and continuing in subsequent weeks, which is attributed to lymphocyte cell death and migration toward the tumor (3). The review by Paganetti outlined that differences in radiosensitivity exist among lymphocyte subpopulations (i.e. B cells appear to be more radiosensitive than T cells, whereas natural killer cells appear to be the most radioresistant).The review indicates that not only the absolute lymphocyte counts are affected by radiation, but also lymphocyte diversity and activity. This is supported by the finding that despite the eventual recovery of the lymphocyte counts, the extent of lymphocyte restoration (i.e. lymphocyte quantity) appears unrelated to survival (i.e. lymphocyte quality) (4).

Numerous studies and reviews have addressed the incidence of RIL and its implications for survival (3). Overall, severe RIL appears to occur in 30-50% of patients with solid tumors, and is most severe after radiotherapy of tumors in the brain, thorax and upper abdomen (5). Multiple meta-analyses have shown the detrimental association of RIL with pathologic response, progression-free survival (PFS) and overall survival (OS), both for specific tumor locations (3, 6–8) and for solid tumors in general (e.g. a pooled hazard ratio [HR] of 1.65, and a 95% confidence interval [CI] of 1.43-1.90 were found for the negative impact of RIL on OS in solid tumors) (5).

Multiple factors have been found to contribute to the occurrence and severity of RIL. Clinical characteristics described in the literature associated with RIL (e.g. lower baseline ALC, older age and worse patient performance score) may correspond to a more fragile reserve or compensation system (5). Other factors such as a higher tumor stage, a larger planning target volume (PTV), more radiotherapy fractions and a higher heart, lung or integral body dose, correspond to a larger proportion of the blood pool (i.e. circulating lymphocytes) being irradiated (4, 7, 9). More recently, the effective dose to immune cells (EDIC) was found to be significantly correlated with severe RIL in esophageal squamous cell carcinoma by Xu et al. and Qiu et al. (10) and in breast cancer by (Chen et al.). The EDIC estimates the dose to immune cells by calculating the radiation dose to circulating blood as a surrogate, with contributions from each blood-containing organ, including the lungs and heart, and large and small blood vessels. Another study confirmed that severe RIL was associated with a higher dose of circulating blood cells. In addition, in a study of hepatocellular carcinoma patients with bone metastases treated with radiotherapy, Chen et al. found that the systemic immune-inflammation index and the neutrophil-to-lymphocyte-ratio were independently correlated with poor prognosis.

A significant finding since the advent of immunotherapy is that a reduction in lymphocyte numbers appears to correlate with decreased effectiveness of lymphocyte-activating immunotherapeutic agents (11, 12). In a meta-analysis by Zhang et al., including 10 cohorts with a total of 1,130 lung cancer patients treated with immunotherapy, RIL was associated with worse PFS (HR 2.05, 95% CI 1.62-2.60) and OS (HR 2.69, 95% CI 2.10-3.43), suggesting the importance of monitoring lymphocyte counts in lung cancer patients undergoing immunotherapy. A study by Pasquier et al. found that the inclusion of at least one tumor-draining lymph node (TDLN) in the clinical target volume was associated with worse PFS in locally advanced non-small cell lung cancer (NSCLC) patients treated with immunotherapy after concurrent chemoradiation therapy. Radiotherapy targeting TDLNs may disrupt the anti-tumor immune response by interfering with the generation of progenitor-exhausted T cells that seed the tumor, resulting in diminished infiltration of CD8+ T cells and decreased expression of T-cell recruiting chemokines.

Identifying patients with an elevated risk of developing RIL is desirable to mitigate this risk and potentially improve oncological outcomes. Such patient selection may help to determine who may benefit most from strategies aimed at avoiding RIL. Multiple prediction models for RIL have been reported in the literature and Van Rossum et al. externally validated two models, developed in lung and esophageal cancer. The PTV-based predictive model yielded better external performance in NSCLC patients when compared to a dosimetry-based model (13). Xu et al. developed and internally validated a machine learning model to predict severe lymphopenia during pelvic radiotherapy in cervical cancer patients. Consequently, these predictive models may assist in identifying patients at increased risk for severe RIL who may benefit from lymphopenia-mitigating strategies, which may ultimately improve survival.

Methods to potentially mitigate lymphopenia are aimed at minimizing unintended radiation exposure to circulating blood and secondary lymphoid organs. This includes avoiding doses to major vessels, the heart, lungs, and lymphocyte-rich organs such as lymph nodes, spleen, and bone marrow. A recent systematic review summarizes the existing literature on dosimetric factors associated with RIL in solid tumors and provides a foundation for potential use in future clinical trials aimed at mitigating RIL risk (14). Since these constraints have not been validated in prospective trials, adhering to the “as low as reasonably achievable” (ALARA) principle for organs at risk remains advisable in current practice. Other methods include reducing the number of radiation fractions (i.e. hypofractionation) or reducing the field size or integral body dose (e.g. with proton-beam therapy or online adaptive [MRI- or CT-based] radiotherapy) (15–17).

In conclusion, RIL is linked to poorer oncologic outcomes in patients with various types of solid tumors. While clinicians may currently have limited awareness of RIL, it is expected to become increasingly important in the coming years with the introduction and advancement of immunotherapy. Future research should determine whether and in which patients’ lymphopenia-mitigating strategies could be beneficial in terms of oncological outcomes.

## Author contributions

PD: Investigation, Project administration, Writing – original draft. SL: Conceptualization, Investigation, Supervision, Writing – review & editing. Pv: Conceptualization, Investigation, Project administration, Supervision, Writing – review & editing.
